# Phase angle as a prognostic indicator of surgical outcomes in patients with gastrointestinal cancer

**DOI:** 10.2478/raon-2023-0060

**Published:** 2023-11-30

**Authors:** Jana Gulin, Ester Ipavic, Denis Mlakar Mastnak, Erik Brecelj, Ibrahim Edhemovic, Nada Rotovnik Kozjek

**Affiliations:** Medical Centre Ljubljana, Ljubljana, Slovenia; Institute of Oncology Ljubljana, Ljubljana, Slovenia; University of Ljubljana, Faculty of Medicine, Ljubljana, Slovenia

**Keywords:** phase angle, colorectal cancer, postoperative complications, malnutrition

## Abstract

**Background:**

In patients with gastrointestinal cancer with planned elective surgery, malnutrition increases the risk of adverse outcomes in the postoperative period. The phase angle, measured by the bioelectrical impedance analysis is an indicator of the metabolic and functional status of the patient. It may be an important prognostic indicator for the clinical outcome of post-surgical treatment in patients with gastrointestinal cancer.

**Patients and methods:**

In this prospective study, 70 patients with gastrointestinal cancer had their phase angles measured by the bioelectrical impedance analysis before the surgery. During the first month after the surgery, we documented the postoperative complications from the patient's records and classified them according to the Clavien Dindo classification of surgical complications. The time of hospitalization was also recorded. The data was statistically analysed in SPSS.

**Results:**

We found a statistically significant difference (p = 0.036) in the average value of phase angles between the group of patients who had postoperative complications (phase angle 5.09°) and the group without postoperative complications (5.64°). We noted a correlating trend of decreasing phase angle values and increasing hospitalization time (Pe R = −0,40, p = 0,001). The phase angle cut-off value (5.5°) was calculated using the ROC curve method, predicting a higher risk of the postoperative complications (p = 0,037) in patients with lower phase angle.

**Conclusions:**

Lower phase angle values before surgery were associated with more complications during the first month after surgery and longer hospitalization time. We found that a phase angle below than 5.5° could serve as a marker that predicts a greater risk of postoperative complications.

## Introduction

Bioelectric Impedance Analysis (BIA) is a technique used to assess body composition and is becoming increasingly established as a tool to assess nutritional status in patients due to its ease of use, low cost, and non-invasiveness.^1,2^ BIA does not directly measure the body composition, but instead measures the drop in voltage of an alternating electric current, as it travels across the body. The phase angle is the quotient of measured resistance and reactance.^1,2,3,4^ It is interpreted as an indicator of membrane integrity and water distribution between the intracellular and extracellular spaces. Phase Angle is used to evaluate body cell mass, which serves as a tool to assess nutritional status in adults and children. Lower phase angle suggests cell degradation with a concomitant decrease in overall cell number with reduced integrity and functional capacity of cell membranes. On the other hand, higher phase angle points to the presence of more functional, intact cell membranes.^5,6,7,8,9^

Malnutrition is a common manifestation of advanced stage cancer and can severely affect the patient morbidity and mortality.^10^ On a cellular level malnutrition is reflected by the diminished integrity of the cell membranes and by altered water distribution throughout the body.^11^ Body composition analysis is therefore an essential tool in assessing nutritional status in cancer patients.^12^ The clinical role of measuring the patient's phase angle is becoming progressively more important. BIA-derived phase angle can help establish guidelines for preventive and supportive measures in patients with malnutrition, as it allows for early identification of high-risk patients with inadequate physiological reserves. The method has previously been shown to have prognostic value in patients with liver cirrhosis, haemodialysis, cancer, HIV/AIDS infection, and lung disease.^5,13,14,15,16,17,18^ For example, patients with stage III or IV colorectal cancer who had a phase angle less than or equal to 5.57° had a median survival of 8.6 months, while those who had a phase angle greater than 5.57° had a median survival of 40.4 months.^14^

The primary objective of this study was to determine if the phase angle can be useful as an independent predictor of post-surgical complications in gastrointestinal cancer patients.

## Patients and methods

### Setting and patients

Our study was a prospective observational study that was performed at the Institute of Oncology Ljubljana, Slovenia, between November 2018 and February 2019. During the study period, BIA was performed on every gastrointestinal cancer patient over 18 years of age that was admitted for elective surgery at the Institute of Oncology Ljubljana and agreed to participate in the study. Exclusion criteria were pregnancy or an implanted pacemaker. The study protocol was approved by the Ministry of Health Medical Ethics Committee and the Institute of Oncology (No. 0120-518/2018/7). Every included patient was fully informed of the study design and signed a written informed consent form.

### Bioelectrical Impedance Analysis (BIA) and other measurements

BIA was performed with a Bodystat (R) Quadscan 4000 machine (Douglas, UK). This phase-sensitive BIA device uses an alternating current of 0.8 mA with frequencies of 5, 50, 100, and 2000 kHz to measure the body impedance. The BIA-derived phase angle was calculated from the impedance at 100 kHz.

Every patient had their phase angle measured the day before surgery and then a week and a month after the surgery. The patients were instructed to abstain from eating and drinking for at least 4 hours prior to the measurement and to abstain from any physical exercise for 8 hours prior to the measurement as well. Two pairs of electrodes were placed on the dorsal side of their right hand and right foot, and they were instructed to lie still in a supine position with no parts of the body touching one another. Clinical data, including gender, age, the exact location of malignant disease, complications in the postoperative course, and hospitalization time were obtained from the hospital information system.

### Clavien Dindo Classification of surgical complications

The patients were categorized in different grades of the Clavien Dindo classification of surgical complications (revised 2004).^19^ The classification uses the degree of most severe pharmacological or surgical treatment needed after surgery, to distinguish between the grades of post-surgical complications. A normal postoperative course without any complications (use of analgesics is considered as normal postoperative course) is classified as degree 0. Degree 1 allows the use of antiemetics, antipyretics, potent analgesics, diuretics, physiotherapy, and treatment of wound infections. Degree 2 additionally includes the use of other drugs, blood transfusion, and total parenteral nutrition. Degree 3 allows surgical, endoscopic, or radiological interventions. Degree 4 includes life-threatening complications that require treatment in an intensive care setting. Fatal complications are classified as a degree 5.

### Statistics

Numeric variables are expressed in terms of their mean values and a 95% confidence interval. Discrete variables are expressed as percentages. The Shapiro-Wilk test was used to check whether the data is normally distributed. The limit for statistical significance was set at p < 0.05. Logistic regression was used to test whether the initial preoperative phase angle, as an independent variable, impacts the odds of post-surgical complications. The results are expressed as quotients. A cut-off value for the phase angle as a predictive factor for post-surgical complications was estimated with a ROC curve, and its sensitivity and specificity were calculated. The statistical analysis was done with the IBM SPSS 23.0 statistical program.

## Results

During the study period (between November 2018 and February 2019), BIA analysis was performed on every gastrointestinal cancer patient planned for elective surgery at the Institute of Oncology Ljubljana that agreed to participate in the study. Seventy patients were recruited. Characteristics of the included patients (column 1), of patients without any complications (column 2) and patients with complications (column 3) are presented in Table 1. The mean age of all patients was 65.0 ± 10.7 years, and 71% were male. Most patients were admitted because of rectal cancer (64.3%). Mean phase angle of all patients was 5.23° ± 2.77°. In total, 52 (74,3%) patients had a complication (Clavien Dindo grade 1–5) after surgery (Table 1 and Table 2).

**TABLE 1.** Characteristics of patients

**Characteristics**	**All patients**	**Patients with no complications (G0)**	**Patients with complications (G1–G5)**	**p-value**
**Gender**				
**Male**	50 (71 %)			
**Female**	20 (29 %)			
**Age (years)^a^**	65.0 (10.7) [62.4–67.6]	61.7 (9.8) [56.9–66.6]	66.1 (10.9) [63.1–69.2]	0.137
**Phase Angle (°)^a^**	5.23 (2.77) [5.0–5.5]	5.64 (0.72) [5.3–6.0]	5.09 (0.98) [4.8–5.4]	0.036*
**Location of the primary tumor (%)**				
**Colon**	17 (24,3)	4 (22.2)	13 (25.0)	
**Rectosigmoid**	2 (2,9)	1 (5.5)	1 (1.9)	
**Rectum**	45 (64,3)	11 (61.1)	34 (65.4)	
**Anus**	1 (1,4)	1 (5.5)	0 (0)	
**Breast**	1 (1,4)	0 (0)	1 (1.9)	
**Ovary**	4 (5,7)	1 (5.5)	3 (5.8)	

a Arithmetic mean (standard deviation) [confidence interval].

* Statistically significant p-value

**TABLE 2.** Mean age and mean phase angle in each Clavien Dindo grade

	**Number of patients (%)**	**Age (years) ^a^**	**Phase angle (°)^a^**
**Grade 0**	18 (26)	61.7 [56.9–66.6]	5.64 [5.28–6.00]
**Grade I**	5 (7)	56.0 [50.2–61.8]	6.22 [5.64–6.80]
**Grade II**	38 (54)	67.1 [63.4–70.7]	4.99 [4.67–5.31]
**Grade III**	4 (6)	62.2 [45.0–79.5]	5.15 [3.41–6.88]
**Grade IV**	5 (7)	72.2 [60.5–83.9]	4.64 [3.77–5.51]
**Grade V**	0 (0)		

a Arithmetic mean (standard deviation) [95% confidence interval].

### Phase angle and the likelihood of complications

**FIGURE 1.**
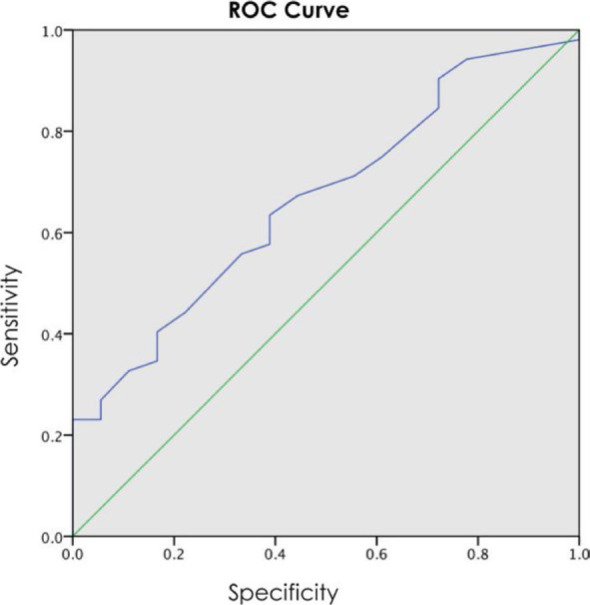
The ROC curve for the phase angle.

The phase angle of patients without a complication was significantly higher than that of the patients with a complication (5.64° ± 0.72° vs. 5.09° ± 0.98°, p = 0.036). Univariate logistic regression analysis showed that the phase angle was associated with the likelihood of a complication (phase angle: odds ratio = 0.48). The odds of a complication in a patient with a phase angle of 5.0 was 3.83, whereas it was only 1.84 in a patient with a phase angle of 6.0. The probabilities of the occurrence of a complication at different phase angles were calculated and are shown in Table 3. The area under the curve (AUC) of the ROC curve for phase angle for the likelihood of a complication is 0.666 (CI: 0.529 – 0.803, p = 0.037), see Figure 1. The determination coefficient (Nagelkerke R square) is 0.104, which means that our model explains 10.4% of the complication likelihood variance. The Hosmer and Lemeshow test value for the model is 0.766.

**TABLE 3.** Odds for developing a complication and probabilities of developing a complication at different values of phase angle

**Phase Angle (°)**	**Odds of a complication**	**Probability of a complication (%)**
4.5	5.532	84.7
5.0	3.831	79.3
5.5	2.651	72.6
6.0	1.837	64.8

### The optimal cut-off value for the phase angle

The cut-off value of phase angle that was derived from the ROC-curve was 5.5° (Figure 1). It yielded a sensitivity of 63% and a specificity of 61% (Table 4). The likelihood of a complication was higher in patients with phase angle below 5.5° than in the patients with phase angle above that value (82.5% vs. 63.3%, Figure 1).

**TABLE 4.** Contingency Table with the sensitivity and a specificity of the phase angle

	**Phase angle < 5,5**	**Phase angle ≥ 5,5**	
**Complication**	33 (true positive)	19 (false negative)	52
**No complication**	7 (false positive)	11 (true negative)	18
	40	30	

## Discussion

The patients with a lower initial phase angle were shown to have a statistically significant higher likelihood of post-surgical complications. The mean phase angle in the group with complications (5,64°) was statistically higher than the mean phase angle in the group without complications (5,09°).

We used univariate logistic regression to calculate the likelihood of a complication at different initial phase angle values. At a phase angle of 5.0°, the odds of a complication are 3.83, and the likelihood of a complication is 79.3%. At a phase angle of 6.0°, the odds of a complication are reduced to 1.84, and the likelihood of a complication is reduced to 64.8%.

We are not aware of any prospective studies investigating the role of phase angle as a prognostic indicator of post-surgical complications in colorectal cancer patients. The retrospective data analysis from 210 elderly patients (aged ≥ 65 years) undergoing gastrectomy showed that preoperative low phase angle predicts a higher risk of postoperative complications.^20^ Similar observations were made in a study among patients admitted to the ICU, where phase angle at admission was shown to be a predictor of 90-day mortality. The mean phase angle of survivors was significantly higher than that of the non-survivor group (5.0° ± 1.3° versus 4.1° ± 1.2°, p < 0.001).^21^ In a study that compared outcomes after cardiothoracic surgery between a group of low phase angle (< 5.38°) patients and a group of normal phase angle patients, the participants from the first group had a higher number of post-operative infections, a larger percentage of them were ventilated for more than 12 hours, and had higher rates of mortality. However, after considering other parameters like sex, age, the type of operative procedure, risk assessment, inflammation activity, hypoalbuminemia, heart failure, time of cardiopulmonary bypass, and time of aortic cross-clamp, the phase angle was found not to be statistically significant in correlation with aforementioned complications. Still, the difference between the groups stayed statistically significant in regard to the hospitalization time and the time spent in the intensive care unit.^22^

In addition, we aimed to find a cut-off phase angle value that can be used to identify high-risk patients that are more likely to have a severe complication after gastrointestinal cancer surgery. Using 5.5° as a cut-off value, we were able to successfully identify these patients in in 67% of the cases with a sensitivity of 63% and a specificity of 61%.

Different phase angle cut-off values have appeared in literature to identify patients with lower physiological reserves who are at risk for increased morbidity and mortality. The proposed PA values in literature are 5.5° for patients newly admitted to the hospital^23^, 4.73° for patients newly diagnosed with head and neck cancer^24^, and 5.2° for patients with various cancers before starting radiotherapy^25^. The phase angle values put forward as a predictor of survival were 4.5° for patients with a non-small cell lung cancer^15^, 5.0° for patients with advanced pancreatic cancer^26^, 5.4° for patients with liver cirrhosis^5^, and 5.57° for patients with colon and rectum cancer^14^.

One of the limitations of this study is a relatively heterogeneous group of patients. The patients included in our study had cancer in different locations and stages in abdomen and they were undergoing different treatment protocols. Additionally, our analysis only took into account the pre-operative value of the phase angle. The patients’ phase angle might have changed in the peri-operative period, especially on account of nutritional support or further medical interventions. Therefore, the value might not have been representative of the patients overall physical state within the entire observed period. The obtained cut-off phase angle value of 5.5° needs to be considered as a tentative value as it was calculated using a specific sample in one population. Further research is needed to identify the cut-off value for different subtypes of colorectal cancer and to evaluate the validity of our obtained cut-off value in distinct clinical settings.

The primary clinical implication of this study is that the phase angle measurement can assist in identifying GI cancer patients with a higher risk of post-operative complications. This could benefit patients that would otherwise not have been identified. Further research is needed to investigate if nutritional or other medical interventions can significantly alter the phase angle and thus affect surgical outcomes.
